# Automatic support control of an upper body exoskeleton — Method and validation using the Stuttgart Exo-Jacket

**DOI:** 10.1017/wtc.2020.1

**Published:** 2020-09-04

**Authors:** Raphael Singer, Christophe Maufroy, Urs Schneider

**Affiliations:** Biomechatronic Systems, Fraunhofer-Gesellschaft, Institute for Manufacturing Engineering and Automation (IPA), Stuttgart, Germany

**Keywords:** Exoskeletons, Human-Robot Interaction, Physical Human-Robot Interactive Controllers, Industry, Control

## Abstract

Although passive occupational exoskeletons alleviate worker physical stresses in demanding postures (e.g., overhead work), they are unsuitable in many other applications because of their lack of flexibility. Active exoskeletons that are able to dynamically adjust the delivered support are required. However, the automatic control of support provided by the exoskeleton is still a largely unsolved challenge in many applications, especially for upper limb occupational exoskeletons, where no practical and reliable approach exists. For this type of exoskeletons, a novel support control approach for lifting and carrying activities is presented here. As an initial step towards a full-fledged automatic support control (ASC), the present article focusses on the functionality of estimating the onset of user’s demand for support. In this way, intuitive behavior should be made possible. The combination of movement and muscle activation signals of the upper limbs is expected to enable high reliability, cost efficiency, and compatibility for use in industrial applications. The functionality consists of two parts: a preprocessing—the motion interpretation—and the support detection itself. Both parts were trained with different subjects, who had to move objects. The functionality was validated both in the cases of (A) an unknown subject performing known tasks and (B) a known subject performing unknown tasks. The functionality showed sound results as it achieved a high accuracy ( 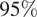 ) in training. In addition, the first validation results showed that this functionality is useful for integration in an appropriately adapted ASC and can then enable comfortable working.

## 1. Introduction

According to latest statistics (e.g., BMAS-BAuA, 2019), musculoskeletal diseases (MSDs, ICD-10 M00–M99) are the main cause for days of absence of work in Germany, with a share of 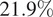 and a yearly tally of approximately 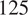 million days. The same situation reigns in Europe (de Kok et al., 2019) and in other industrialized countries. Occurrence of MSDs is in many cases caused by working in poor ergonomic postures, or excessive physical strain on specific body parts (mostly back and shoulder regions), or both.

In this context, *occupational exoskeletons*, that is body-worn assistive devices aimed at relieving physical load on the worker’s body, are gaining increasing importance as potential solutions to reduce fatigue and alleviate the risk of injuries during manual handling or overhead work. Passive ones make a considerable contribution to this, particularly in the case of overhead work, but are rather unsuitable for lifting and carrying activities and many other applications due to the lack of flexibility in terms of adapting the support. In contrast, active exoskeletons can provide much higher flexibility and transparency if the level of support can be adequately adjusted depending on the execution of the task at hand. Active exoskeleton systems for assisting, lifting, and carrying heavy objects rely mainly on one of two following approaches. In the first one, handling of the object is achieved by the exoskeleton, which is equipped with hooks or grippers. The exoskeleton structure usually extends to the ground, which provides a parallel mechanical pathway for the transmission of the weight of carried object, potentially enabling to manipulate weights over normal human carrying capacity, see, for example, Sarcos (2019).

Regarding support control, this situation is close to robot teleoperation and control approaches based on the measure of the exoskeleton–user interaction forces can be applied (Miller and Rosen, 2010). The main drawbacks of the approach are the large number of actuators needed (with consequences on system weight, price, size, and speed) and the limitations regarding grasping due to the robotic gripper (e.g., dexterity and handling of soft objects).

For these reasons, another category of active exoskeletons, directly anchored to the human body and similar in structure to passive exoskeletons, has been increasingly investigated in recent years. These systems enable direct human–object contact, enabling to fully leverage hand dexterity which is needed in many cases, and ensure sufficient grasping flexibility and handling speed. Their goal is to prevent MSDs by relieving the excessive loads acting on specific body parts while lifting and carrying objects within normal human capability. It is therefore to be expected that the second approach will be highly demanded. However, control of support is not trivial as the exoskeleton structure cannot directly sense the load a person is lifting or carrying with his hands. This is the fundamental task control problem of direct human–object contact exoskeletons, for which solutions are sought in the field of body-worn devices.

Approaches for prosthesis (Fan and Li, 2010; Young et al., 2014) and lower limb exoskeletons (Yan et al., 2015) deal with the topic of the recognition of motion and the regulation of actuators on humans. Although they are a good source of inspiration concerning sensors and methods, they are not directly applicable.

Regarding upper body exoskeletons, different approaches have been proposed so far, such as trigger buttons placed at the fingers that are pressed on demand as in Abbruzzese et al. (2011). Although these indicate the direct user request, manipulation of objects is impeded and the handling of the button is for the most part cumbersome. Other approaches use gloves with force sensors, or flexion sensors, or both, to estimate whether objects are hold in a hand (Nilsson et al., 2012; Otten et al., 2016; Stadler et al. 2016; Stelzer et al., 2016). These concepts, however, block the tactile sense and reduce dexterity.

Manufacturing a highly sensorized glove sufficiently robust and economical for manual handling of applications also present technical challenges toward practical use. The approach used by the Innophys muscle suit, which is controlled by blowing into a tube or touch a surface using one’s chin (exoskeletonreport, 2020), gives more freedom, but its suitability for industrial long-term use is questionable. Toxiri et al. (2017) proposed an assistive strategy based on inertial sensors and sensorized shoes measuring foot pressure. Although the approach is straightforward, the distinction between dynamic forces and the picking up of loads is difficult, especially if this is less than approximately 10 kg. In addition, the communication with the shoe module relies either on a potentially error-prone wireless or on an impractical wired connection.

Direct motion control via surface electromyography (sEMG) is a concept often used in medical assistance devices (Rosen et al., 2001; Kiguchi et al., 2005; Kiguchi and Hayashi, 2012; Lenzi et al., 2012; Ebrahimi et al., 2014; Li et al., 2014; McBean and Narendran, 2016). However, due to the large number of sensors and time-consuming calibration phases, the transfer of this technology to occupational exoskeletons for industrial applications has proved to be hardly possible.

Maufroy and Bargmann (2018) investigated the use of arrays of sEMG on the forearms in order not to directly control the movement, but to detect grasping and identify objects held in the hand. As an alternative to sEMG sensors, muscle circumference or stiffness sensors that are appealing as they can be worn over clothing, have also been investigated (Khan et al., 2014; Kim et al., 2014). Although concepts based on muscle contraction information are promising, they often lack robustness because they rely only on one type of signal that can occasionally give misleading outputs.

Kinematic information of limb motion provides another promising type of signal based on which the users’ movements can be interpreted and support can be given as situation demands (e.g., lifting of a box). Theiss et al. (2016) and Malaisé et al. (2019) deal here with gesture or activity recognition, respectively, and Stančić et al. (2017) deals with hand gestures that are interpreted by hidden Markov models (HMM), but all of them not in the context of automatic support control (ASC).

Despite many promising approaches, the problem of ASC is still not solved satisfactorily. This article presents a concept for ASC applied for lifting and carrying activities aiming at high reliability and practicality with cost efficiency by combining processed limb motion information with simple muscle activation signals. The focus here is on the key functionality of estimating the onset of user’s demand for support. The “Methods” section presents the exoskeleton platform used to collect the experimental data and then describes more in detail the investigated approach of the ASC algorithms, as well as the training and validation methods for the motion interpretation. Results after the training and with respect to the validation of the robustness of the proposed approach (using an unknown subject and situations) are presented in “Results” section, while a conclusion including suggestions for future research is given in “Conclusion and Future Work” section.

## 2. Methods

### 2.1. Exoskeleton-platform Stuttgart Exo-Jacket 2.0

**Figure 1.**
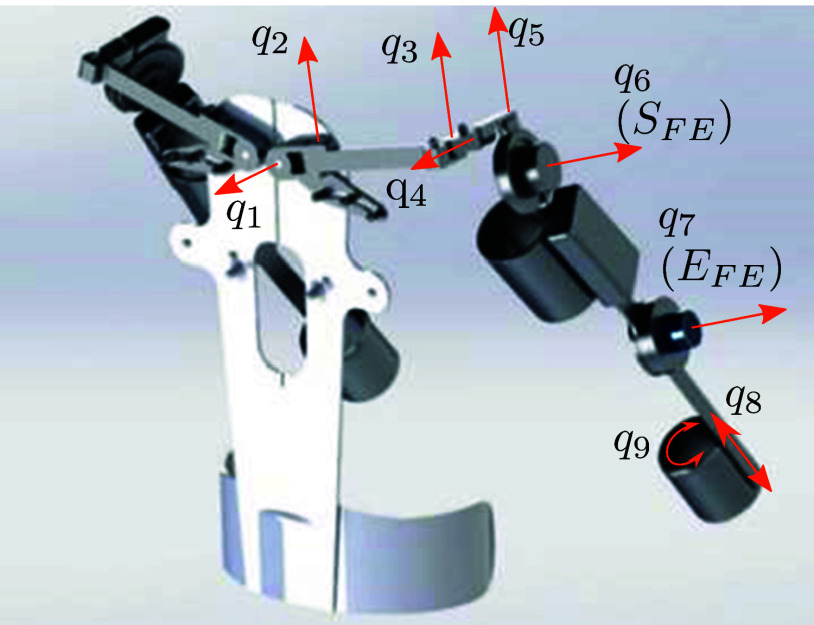
CAD model of Stuttgart Exo-Jacket 2 (SEJ2) showing the nine degrees of freedom 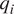 , with the shoulder joint 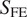 and elbow joint 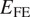 actuated.

The experiments and data collection were carried out with the upper limb active exoskeleton platform Stuttgart Exo-Jacket (SEJ) 2. Compared to the SEJ1 (Ebrahimi, 2017; Ebrahimi et al., 2017), a new shoulder mechanism was designed and additional passive degrees of freedom (DoF) were added to improve support and reduce misalignment (cf. Figure 1 and Tröster et al., 2018). Two of the nine DoF are actuated by flat electronically commutated motors combined with harmonic gearing, while the remaining ones are passive. The shoulder actuator ( 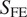 ) has a nominal torque of 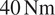 and maximal angular speed of 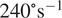 , the elbow actuator ( 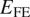 ) 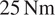 and 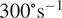 , respectively. These allow accelerations and speeds sufficient to match the dynamics of human arm movements in usual object handling applications. Thanks to the actuators’ high-power density and the simple mechanical design, the exoskeleton is light enough to be worn by humans and not significantly impeding them in their activities.

### 2.2. Control Architecture

**Figure 2.**
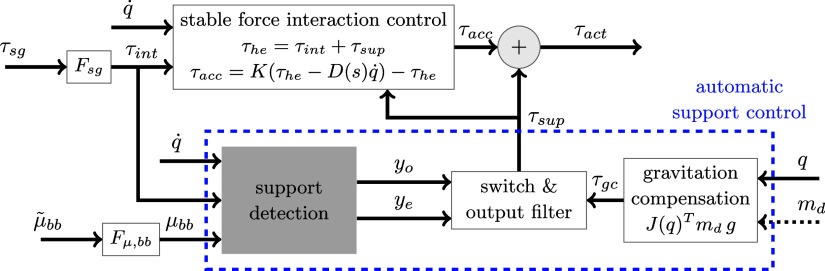
The Stuttgart Exo-Jacket 2 (SEJ2) control architecture composed of stable force interaction control (SFIC) and the automatic support control (ASC). The SFIC computes the interaction torque 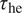 resulting from the human muscle force acting on the exoskeleton from filtered strain gauge torque 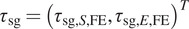 . The acceleration torque 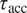 is 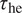 amplified by 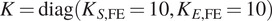 and stabilized with 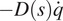 where 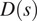 is a low pass filter with gain 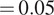 , 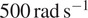 and 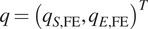 is the shoulder and elbow joint angles vector. The ASC outputs the support torque 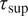 based on the gravitation compensation torque 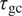 and the detection of onset 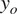 and ending 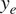 of support, estimated by support detection module (SD). SD takes 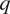 , 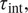 and 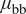 , the filtered biceps brachii muscle activation 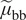 . 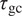 for both active joints is computed from the support preset 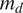 (in 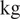 ), the gravitation vector 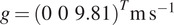 and the Jacobian 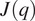 between 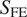 and the human hand. 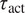 is the desired torque sent to the actuators. 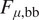 , 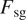 are filters.

The SEJ2 control architecture represented in Figure 2 is made of two parts: stable force interaction control (SFIC) and the ASC. The SFIC enables a transparent behavior for the user by adjusting the generated torque based on the measured user–exoskeleton interaction forces. The ASC calculates the required torques for each joint based on joint configuration and support preset 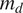 , and adds these when estimated by the support detection. The estimation of onset and ending of support is the task of the support detection module (SD). This module is a key functionality of the ASC and the focus of the present article.

### 2.3. Sensors and Data Acquisition

SEJ2 active joints are with encoders and strain gauges in order to measure angles 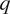 and torques 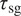 . To request support the SEJ2 has one push button per hand, which is used in the following as a subjective feedback. A working ASC can make these buttons obsolete. The activity of the biceps brachii muscle 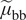 was selected as simple muscle activation signal to complement limb motion information. The objective was to add as little additional sensors to the system as possible in order to save costs and avoid cumbersome donn−/doffing. The biceps brachii is particularly suitable for this, as it is largely involved in lifting activities and easily accessible in practice. It is measured for each arm using a low-cost EMG sensor with threefold dry electrodes (type Gravity^©^ from DFRobot/OYMotion). The sensor contains an internal amplifier with factor 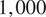 and filter electronics providing outputs in the range of 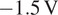 to 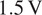 .

All sensors are sampled at a rate of 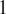 kHz using the rapid prototyping system MicroLabBox^©^ from dSPACE. The strain gauge signals are filtered with the filter 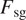 (low pass 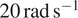 ) and the sEMG signals with the filter 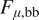 (notch filter 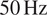 , mean adaption low-pass filter 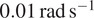 , envelope low-pass filter 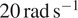 , normalization with maximum).

### 2.4. Support Detection Module

**Figure 3.**
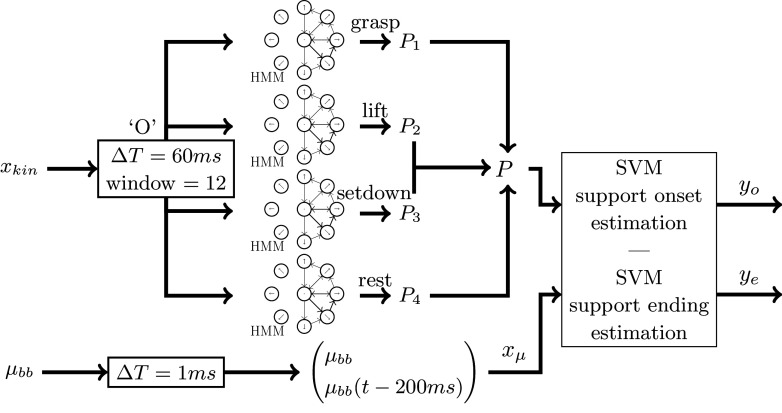
Overview of the proposed support detection approach. Preprocessing: Interpretation of the arm motion using the kinematic variables 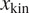 by four hidden Markov models. Classification layer: Merge likelihoods 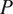 with 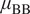 and its delay and estimate onset and ending of support using support vector machines classification.

The support detection module is based on two main inputs in order to achieve a reliable behavior: arm motion 
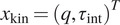
 and muscle activation 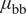 (cf. Figure 3). The torque 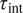 is a measure for the acceleration of the arm motion. The arm motion is preprocessed with a motion interpretation module using HMM to generate likelihoods for predefined motion primitives, for example, grasping or lifting an object (see section “Motion interpretation—concept, measurement, training, and test”).

The support detection is taking the output likelihoods of the motion interpretation and combines it with the muscle activation signal. This layer uses classifiers (support vector machines [SVM]) to estimate the onset and ending of the user’s need for support. This article is focused on the support onset estimation (see section “Support onset estimation—measurements and SVM training procedure”). Support ending estimation would work in a similar way but it is not the scope of the article.

**Figure 4.**
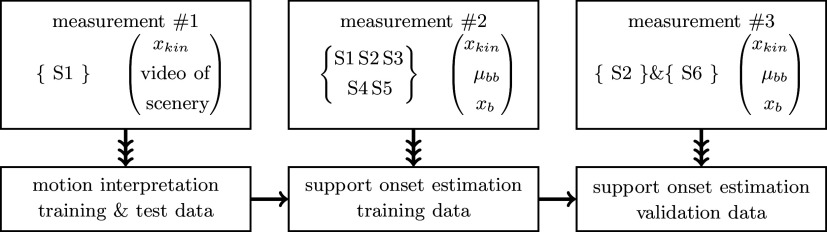
Collection of the training, test and validation data, where 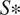 denotes the subject’s number. The motion interpretation is trained and tested with 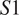 . The training of the support onset estimation is performed with five subjects and validated with 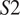 and previously not used subject 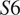 . 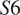 had to perform the tasks which have been performed in training as well, 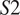 performed other tasks than for training. The reference 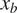 is the button signal (see section “Support onset estimation—measurements and SVM training procedure”).

For motion interpretation and support detection, measurements with different subjects are taken to obtain training and validation data, as represented in Figure 4.

**Figure 5.**
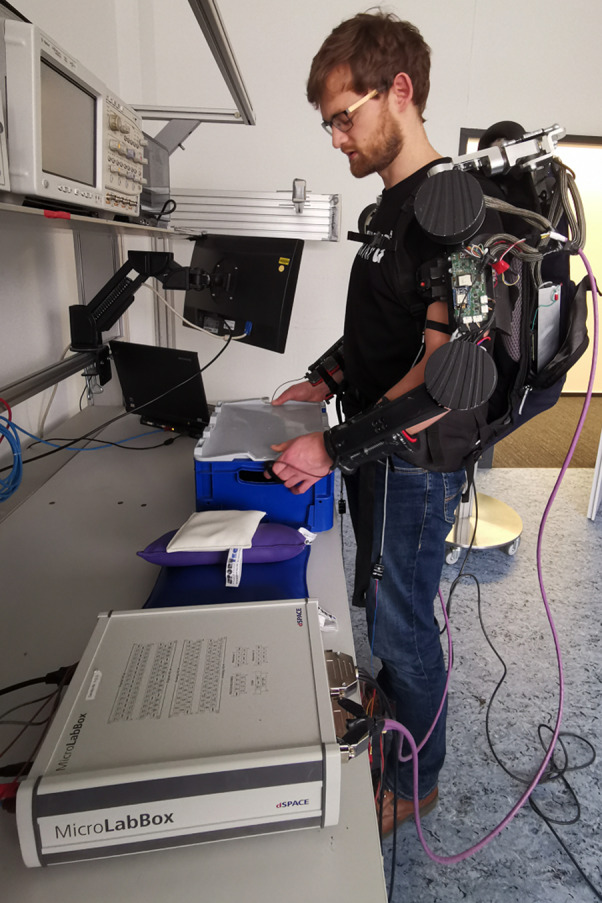
Experimental setup used for data collection, here showing one of the subjects grasping the box to lift.

### 2.5. Motion Interpretation—Concept, Measurement, Training, and Test

Compared to other tasks (like for instance assembly) the process of picking-up objects is of low motion variance. Therefore, it is possible to isolate different motion primitives of box lifting movements. The motion primitives selected here are “grasp,” “lift,” “set-down,” and “rest.” For the development of the motion interpretation module, training and test data of 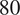 grasps, lifts, set downs, and rests each were recorded with one subject. The data was labeled with one of these four motion primitives. Each input vector 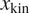 mapped to a distinct observation, that is a number. Each of the four signals of 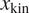 is divided into five sections for this purpose, resulting in a set of 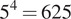 distinct observations. The four HMMs ( 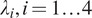 ) have nine hidden states and are trained using 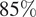 of the measurement data with the Baum–Welch algorithm (cf. Barber, 2012). The HMMs have been implemented using a sample time of 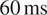 and a sequence length of 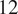 data points. This observation sequence 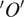 is fed to each of the HMMs. The outputs of the HMMs are the logarithms of the likelihood ( 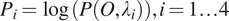 ) that the observation belongs to the HMM.

The motion interpretation module is tested using the remaining 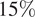 of the measurement data. The maximum of 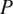 is considered as the output of the motion interpretation and compared with the actual label. Table 1 shows the results in the form of a confusion matrix. Overall, an accuracy of 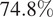 is achieved. In particular, lifts are classified in 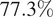 of the cases correctly and in 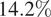 of the cases as grasps (i.e., 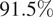 combined). From this perspective, it is an useful contribution to the ASC. However, the achieved accuracy is far from sufficient for working tasks in industry. Hence, the concept is combined with the muscle activation signal 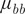 .

**Table 1.** Confusion matrix for the motion interpretation with test data [in 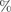 ].

	Estimated			
Actual	Rest	Grasp	Lift	Set-down
Rest	93.6	1.8	0.6	4.0
Grasp	36.1	53.5	10.3	0.0
Lift	4.0	14.2	77.3	4.5
Set-down	12.9	6.2	20.1	60.8

### 2.6. Support Onset Estimation—Measurements and SVM Training Procedure

**Figure 6.**
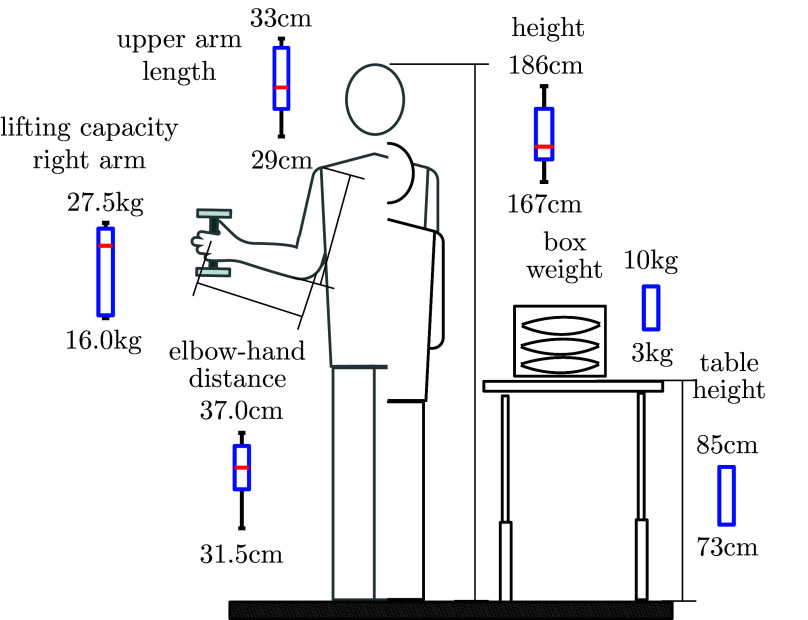
Survey of experiment showing box plots of the anatomical dimensions and muscle strength indicator for the five subjects used for training. The basic setup of experiment with table and box with weights is depicted on the right.

Training and test data were recorded with five subjects (cf. Figure 4 for survey of measurements), among them the subject used for the development of the motion interpretation module. Subjects were selected who showed significant variations of muscle strength (measured as maximum lifting capacity) and anatomic dimensions (e.g., arm length and body height). The maximum lifting capacity has been defined as the maximum weight a person can hold with one hand for 3 s. Data with one additional subject was collected for subsequent validation. All subjects are male and right-handed. In Figure 6 some relevant properties of the subjects are summarized.

Two different table heights (73 and 81 cm) combined with two different box weights (3 and 8 kg) and a pseudo-lift are defined. For the pseudo-lift, the subject is instructed to perform a motion similar to a lift, however, without actually grasping and lifting the box. Altogether, 6 combinations with 20 repetitions each were performed by each training subject. The recording of training data started for each subject with the first table height and the first weight, then iterated over the different weights and proceeded with the next table height. The subjects were instructed to push the SEJ2 support request button in their hand as long as support was desired, that is between lifting up and placing the box back on the table. For the pseudo-lift no button was pressed. The signals from the push buttons were recorded together with the arm motion and muscle activation signals. These were used as reference for the training of the support onset estimation. Each data contains the vector 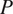 , 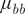 , the button signal 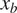 and 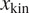 . An additional signal delayed by 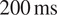 was generated from 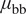 and combined with the signal itself as 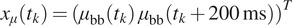 , ( 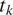 is a sample).

The data were processed with the Matlab^©^ Statistics and Machine Learning™ toolbox using the support vector machine training. At first, the time points 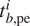 where the button 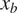 has a positive edge have been selected. Theoretically, only these few points could be handed over to SVM training as the “onset” labeled class. However, their share in the total number of points is so small that there would not be enough information for a well-founded SVM training. Furthermore, at these points, very often there is no or only a slight change in the signals of muscle strength 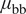 or the interpretation of movement 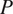 , which often makes an estimation of the onset impossible. In contrast to this, a time interval after 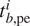 is now manually selected. For each repetition, it should contain the section in which signal changes occur, that are important for the onset estimations. Since these sections vary in time depending on person and repetition, this is defined sufficiently large. The interval is defined as 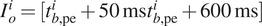 and all points in it belong to the “onset” class. The remaining ones belong to the “default” class. Since training time increases super-linearly with the number of samples, the training data is reduced by downsampling to 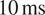 . In addition, only one-third of the data points are randomly selected. An optimization procedure was performed with different kernel functions (linear, quadratic, cubic, and Gaussian), kernel scales, and box constraints (i.e., tolerance for outliers). For this procedure 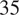 -fold cross validation has been applied to avoid over-fitting.

## 3. Results

### 3.1. Results with Training Data

**Figure 7.**
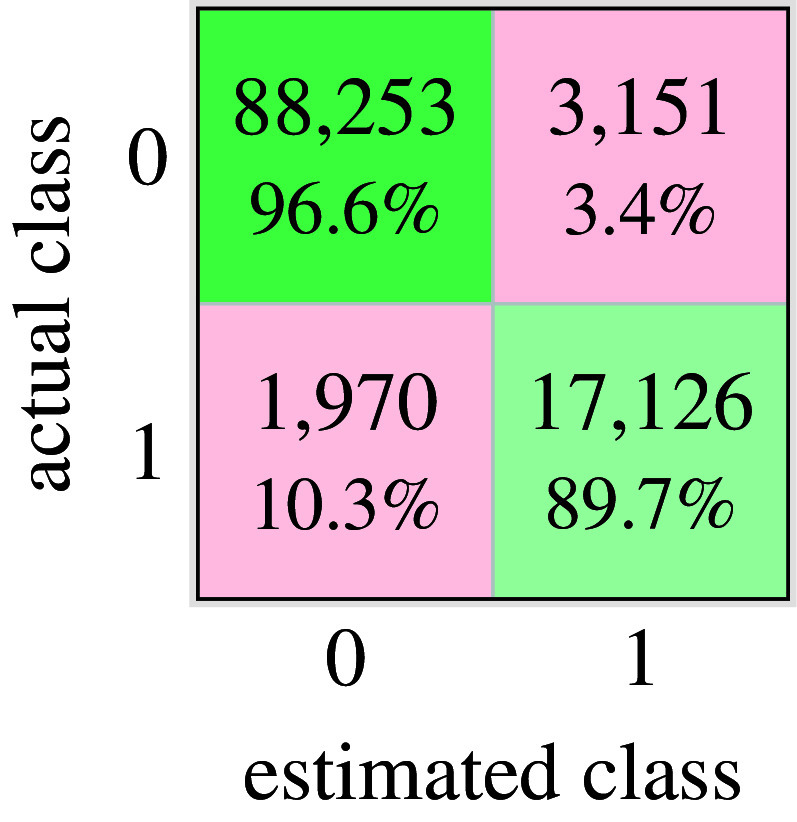
Confusion matrix of cross-validation of training data. Accuracy: 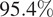 , true negatives: 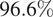 , true positives: 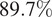 , false negatives: 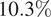 , false positives: 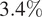 . Class 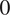 : 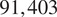 samples, Class 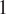 : 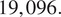

The optimization results in an SVM with Gaussian kernel, a scale of 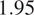 , a box constraint of 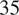 and consists of 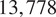 support vectors. The performances are described in the confusion matrix of Figure 7. Accuracy reached 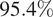 . Here, attention was paid to a small false positive rate ( 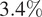 ) at the expense of a higher true negative rate ( 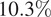 ) by changing the misclassification cost matrix. It is more important to avoid unwanted support onset estimates than missing necessary activations. In the first case, the user’s movements would be disturbed by forces while not carrying any objects, which could lead to safety risks. While in the second case, the user would carry an object and receive no support, which would only lead to unpleasant behavior, as the user would have to carry the weight completely by himself.

In order to evaluate the effect of the selected combination of limb motion and muscle activation signals, a cross-check was performed. In this test, only 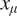 was applied and not the 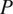 vector, using the same SVM optimization procedure as before. The accuracy did not exceed 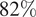 , with a false positive rate of more than 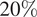 and a true positive rate of less than 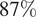 , which emphasizes that the approach of combining limb motion and muscle activation is reasonable as it reaches a considerably higher accuracy.

### 3.2. Results with Validation Data

**Figure 8.**
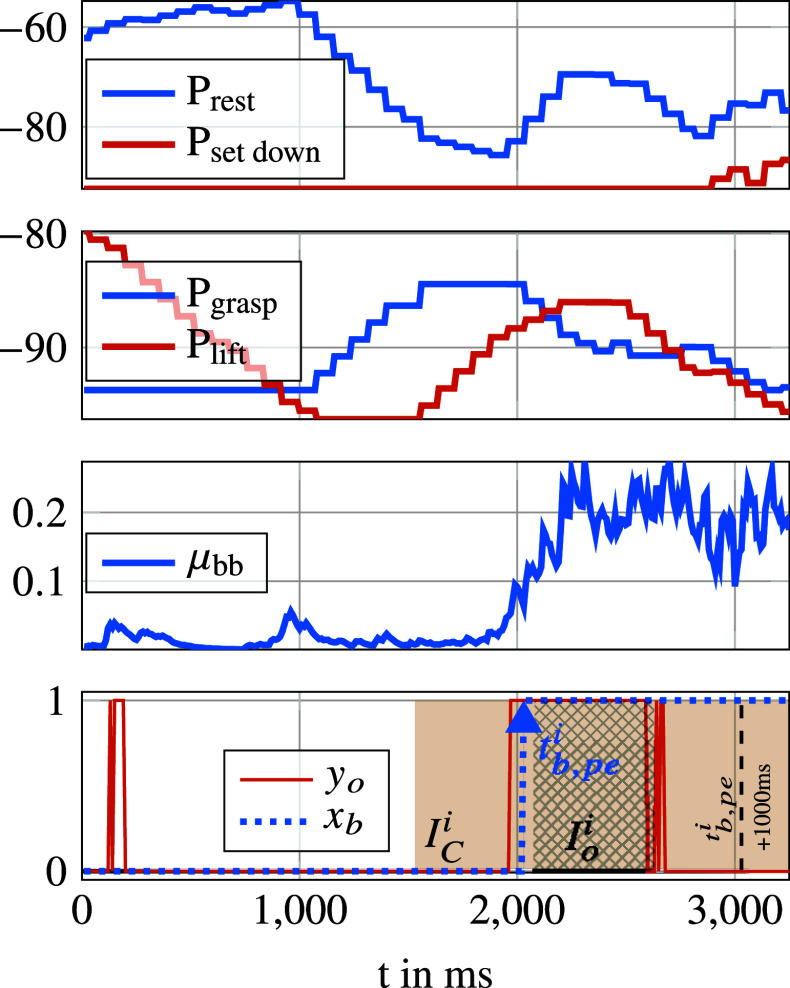
Example result of support detection illustrating unwanted support onset estimations at about 100 ms, support onset estimations beginning shortly before 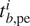 and ending at about 600 ms after 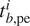 .

For validation, ASC function is tested (A) with a subject who was not involved in the training using the same scenarios (weights and heights) as in the training and (B) with a subject who was involved in the training but with scenarios that did not occur in the training procedure. Beside 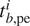 , the points in time 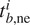 are introduced where the button signal 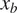 has a negative edge, and as well the corresponding interval 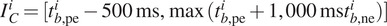 , while 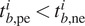 . 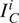 contains the *i* ^th^ repetition. The ASC performance obtained with the validation data are analyzed based on the following four metrics, illustrated in Figure 8.

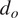

The measure of premature or delayed onset estimation. Defined as the time difference between 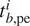 and an occurrence of 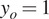 in the corresponding 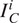 . The greater the proportion of 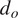 , which lies in 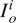 , the better the result. Since similar input signal characteristics are assumed, small temporal fluctuations in the outputs indicate a good quality of the module.

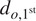

A special case of 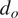 that indicates the first occurrence of 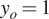 in the corresponding 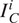 . The closer the distribution of 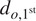 to the left bound of 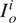 , the better the result. This metric indicates inter alia how quickly the ASC can react.

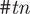

Number of demanded activations (positive edge 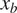 ) without any detection in the corresponding 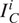 . The smaller the number the better.

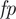

Number of intervals of coherent false support onset estimation points, that is if 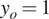 outside of any 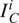 . The smaller the number the better.

#### 3.2.1. Results for unknown subject with scenarios used in training (A)

The data recorded with a subject not involved before in the training process performing the same tasks as the subjects in the training are analyzed.

**Figure 9.**
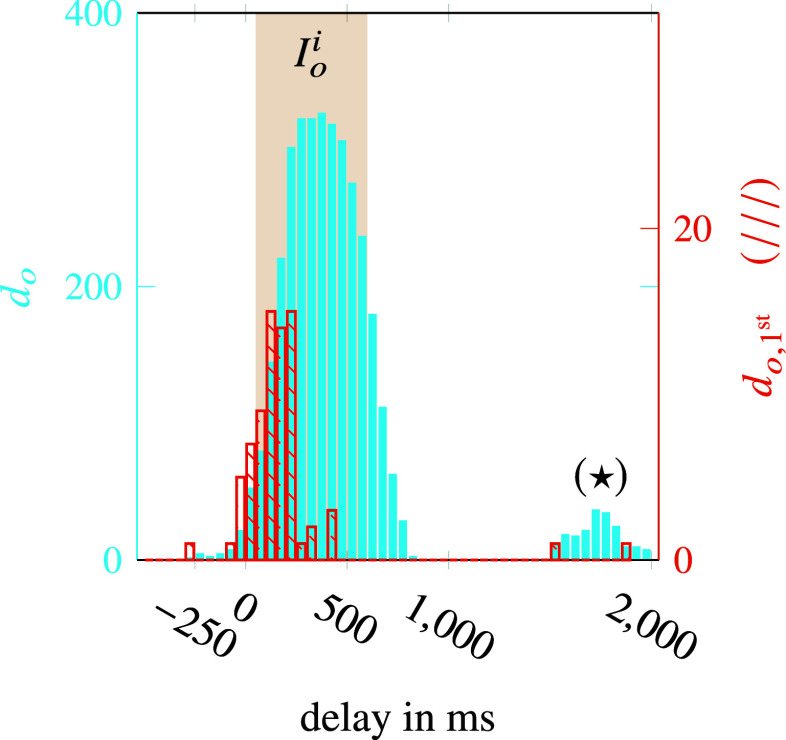
Histogram of onset support estimation validation. Analysis for unknown subject showing the distribution of 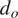 (filled) and 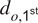 (hatched).

The distribution 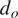 is depicted in the form of a histogram in Figure 9. The majority of onset estimations occurred within 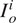 and showed a compact behavior, that is only small fluctuations. However, the smaller peak with a delay of 1,800 ms (cf. 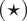 in Figure 9) deviates significantly. If these points are subsequent onset estimations (i.e., not the first ones) in their interval 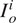 , they hardly pose a problem, since ASC can already react to its predecessors. However, if they are first onset estimations (i.e., without a predecessor) the ASC obviously cannot react earlier.

Therefore, the histogram of 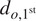 in Figure 9 is considered. It can be observed that on this right side, only two estimates occur and only a small amount ( 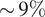 ) has a delay of more than 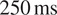 , which is unpleasant but not critical. The majority of first onset estimations is in the left half of 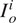 (approximately about 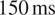 ). Although this delay is of course still perceptible to the wearer, it is already acceptable at this early stage of development and has potential for improvement. However, of the 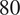 demanded user activations, 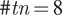 times no activation at all occurred, which with 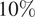 corresponds approximately to the true negative value of the training results and, although not safety-critical, must be improved for an industrial application. More critical are the 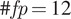 intervals from a total of 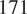 support onset estimates outside of any 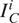 . Cumulatively, these represent only 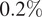 of the total time, but as mentioned previously unwanted trigger of the support activation could lead to safety issues.

#### 3.2.2. Results for known subject with new scenarios not used in training (B)

The data recorded with a subject already involved in the training process performing new scenarios (cf. Table 2) are analyzed.

**Table 2.** Validation procedure with unknown table heights and weights combinations.

	73 cm	77 cm	81 cm	85 cm
3 kg				10×
6 kg	10×	10×	10×	10×
10 kg			10×	
Pseudo-lift				10×

**Figure 10.**
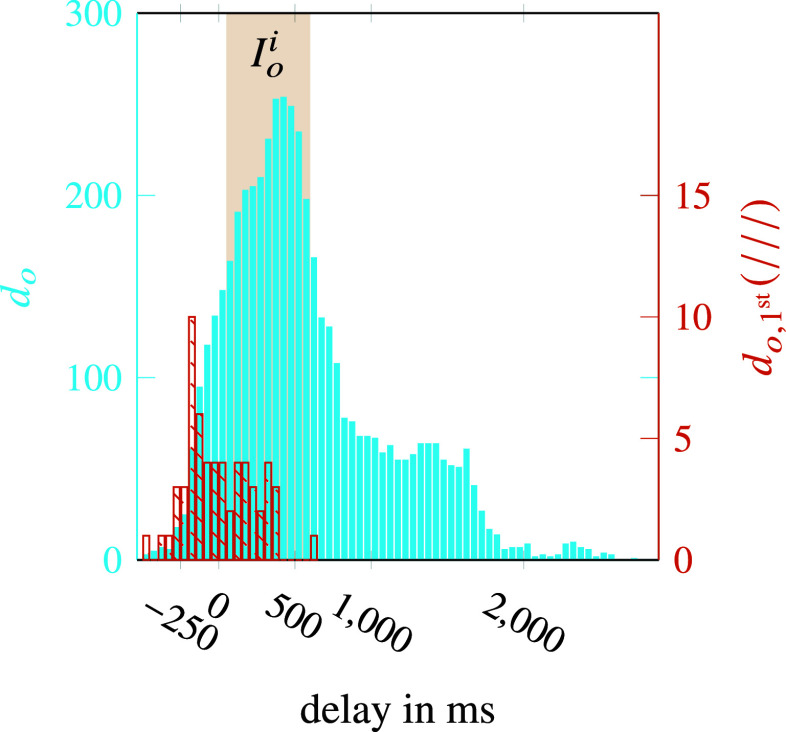
Histogram of onset support estimation validation. Analysis for known subject and unknown scenarios showing the distribution of 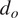 (filled) and 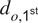 (hatched).

The distribution of 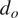 is depicted in Figure 10. In contrast to (A), it is noticeable that the distribution is wider and a large part is outside 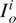 . In order to assess the usability 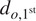 must be included. This shows that the first activation tends to occur earlier than in case (A). While 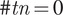 , which is of course positive, 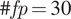 intervals of coherent unwanted onset estimation points occurred. In summary, the module tends to produce more premature onset estimations for the unknown scenarios ( 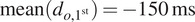 ).

Examining the different situations in detail shows that these generally deliver acceptable results regarding the metrics. One of them ( 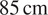 , 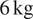 ) with 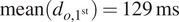 even represents a particularly good result. It turns out that the situation with a table height of 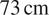 is the main contributor to the poorer result as it contains the majority of 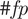 ( 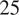 ) and tends to be very premature with 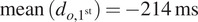 . An explanation for the observations can be the differences in the execution of the arm trajectories. It has been observed that they vary strongly with different table heights, not only in length but as well in shape, since the box handle has to be grasped from different directions. It is reasonable to assume that the motion interpretation does not work robustly enough for the different motions associated with different table heights and users.

## 4. Conclusion and Future Work

An ASC concept based on arm motion and muscle activation information was presented with a special focus on the onset of user’s support demand. Arm motion is processed in four different HMMs outputting the likelihood of different motion types while sEMG sensors capture muscle activation. The likelihoods of motion primitives, the muscle activation signal with its delay and the SEJ2 support trigger button as a reference signal, are combined to record training and validation data for an SVM. Cross-validated results of training data achieved an accuracy of 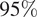 . With this preliminary test run, which only required five training subjects, it was shown that the concept offers a basis for use in an industrial application, since it already showed an acceptable level of security (very few false estimated support onsets of not demanded support) and a low frustration potential (few not detected user demanded activations and a temporal deviation of support activation, most of which is suitable for comfortable working).

The calculation including 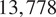 support vectors requires a high computing power, which can be reduced for the purpose of the embedded real-time implementation by limiting the number of support vectors. It has been investigated for instance that reducing the SVM algorithm to 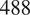 support vectors by allowing more outliers (lower box constraint value) alters the accuracy only slightly ( 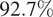 ).

Nevertheless, the ASC function has to be further improved in order to leverage accuracy and improve robustness. Therefore, more training subjects have to be included performing more repetitions in more different situations. For the motion interpretation the manual labeling should be automated. In order to use less computing power alternative concepts other than HMM (wavelets and neural networks) should be tested. Furthermore, abilities should be added to adjust for different anatomies of humans. Integration of inertial measurement units to augment or replace the encoders could easily enable a spatial acquisition of motion, as it is currently only two-dimensional.

Since the upper arms must not be covered when using sEMG sensors for the biceps brachii, either different sensor locations (Maufroy and Bargmann, 2018) or muscle stiffness or circumference sensors are under consideration to allow for long-sleeved clothing.

Further potential for optimization lies in the addition of a validation function, which observes which arm motions and muscle activations an ASC output leads to. Incorrect activations would, for example, lead to the arm being pushed upwards by the exoskeleton. Since no load is carried in the hands, this would either lead to a rapid upward motion of the arms or to strong arm resistance. Both could be detected by suitable sensor combinations, intercepted by the ASC and stopped quickly.

## Data Availability

Data is not available online for this article.
